# Coronary Thermodilution Waveforms After Acute Reperfused ST‐Segment–Elevation Myocardial Infarction: Relation to Microvascular Obstruction and Prognosis

**DOI:** 10.1161/JAHA.118.008957

**Published:** 2018-08-04

**Authors:** Shu Ning Yew, David Carrick, David Corcoran, Nadeem Ahmed, Jaclyn Carberry, Vannesa Teng Yue May, Margaret McEntegart, Mark C. Petrie, Hany Eteiba, Mitchell Lindsay, Stuart Hood, Stuart Watkins, Andrew Davie, Ahmed Mahrous, Ify Mordi, Ian Ford, Keith G. Oldroyd, Colin Berry

**Affiliations:** ^1^ British Heart Foundation Glasgow Cardiovascular Research Centre Institute of Cardiovascular and Medical Sciences University of Glasgow United Kingdom; ^2^ Robertson Centre for Biostatistics University of Glasgow United Kingdom; ^3^ West of Scotland Heart and Lung Centre Golden Jubilee National Hospital Glasgow United Kingdom

**Keywords:** magnetic resonance imaging, myocardial infarction, pathophysiology, Magnetic Resonance Imaging (MRI), Percutaneous Coronary Intervention, Pathophysiology, Coronary Artery Disease, Myocardial Infarction

## Abstract

**Background:**

Invasive measures of microvascular resistance in the culprit coronary artery have potential for risk stratification in acute ST‐segment–elevation myocardial infarction. We aimed to investigate the pathological and prognostic significance of coronary thermodilution waveforms using a diagnostic guidewire.

**Methods and Results:**

Coronary thermodilution was measured at the end of percutaneous coronary intervention, (PCI) and contrast‐enhanced cardiac magnetic resonance imaging (MRI) was intended on day 2 and 6 months later to assess left ventricular (LV) function and pathology. All‐cause death or first heart failure hospitalization was a pre‐specified outcome (median follow‐up duration 1469 days). Thermodilution recordings underwent core laboratory assessment. A total of 278 patients with acute ST‐segment elevation myocardial infarction EMI (72% male, 59±11 years) had coronary thermodilution measurements classified as narrow unimodal (n=143 [51%]), wide unimodal (n=100 [36%]), or bimodal (n=35 [13%]). Microvascular obstruction and myocardial hemorrhage were associated with the thermodilution waveform pattern (*P*=0.007 and 0.011, respectively), and both pathologies were more prevalent in patients with a bimodal morphology. On multivariate analysis with baseline characteristics, thermodilution waveform status was a multivariable associate of microvascular obstruction (odds ratio [95% confidence interval]=5.29 [1.73, 16.22];, *P*=0.004) and myocardial hemorrhage (3.45 [1.16, 10.26]; *P*=0.026), but the relationship was not significant when index of microvascular resistance (IMR) >40 or change in index of microvascular resistance (5 per unit) was included. However, a bimodal thermodilution waveform was independently associated with all‐cause death and hospitalization for heart failure (odds ratio [95% confidence interval]=2.70 [1.10, 6.63]; *P*=0.031), independent of index of microvascular resistance>40, ST‐segment resolution, and TIMI (Thrombolysis in Myocardial Infarction) Myocardial Perfusion Grade.

**Conclusions:**

The thermodilution waveform in the culprit coronary artery is a biomarker of prognosis and may be useful for risk stratification immediately after reperfusion therapy.

**Clinical Perspective**

**What Is New?**

- In acute ST‐segment–elevation myocardial infarction survivors, coronary thermodilution waveforms depicted using a diagnostic guidewire at the end of percutaneous coronary intervention provides information that is linked with microvascular injury.
- A bimodal thermodilution waveform is independently associated with adverse clinical outcomes, including all‐cause death and heart failure in the longer term.
- A bimodal thermodilution waveform is a biomarker for prognostication in survivors of acute ST‐segment–elevation myocardial infarction.

**What Are the Clinical Implications?**

- Risk assessment of failed reperfusion in patients undergoing emergency percutaneous coronary intervention for acute ST‐segment–elevation myocardial infarction is challenging.
- Use of a diagnostic guidewire at the end of the percutaneous coronary intervention has emerging clinical utility for risk assessment.
- Classification of the coronary thermodilution waveform categorization is a novel approach to identify at‐risk subgroups that has the potential to translate into real‐world practice merits assessment.

Percutaneous coronary intervention (PCI) for acute ST‐elevation myocardial infarction (STEMI) routinely leads to successful coronary reperfusion.^1^, ^2^ However, failed myocardial reperfusion, depicted by microvascular obstruction on contrast‐enhanced cardiac magnetic resonance (CMR) imaging, occurs in more than half of these patients,^3^, ^4^ implicating an adverse long‐term prognosis.^5^, ^6^

Microvascular obstruction usually passes undetected in clinical practice. Standard measures of myocardial reperfusion, including TIMI (Thrombolysis in Myocardial Infarction) Myocardial Perfusion Grade and corrected frame count, or ST‐segment resolution on the ECG, lack sensitivity and reproducibility.^6^, ^7^, ^8^, ^9^ CMR is the reference noninvasive technique for detection of microvascular pathology^3^, ^5^, ^10^; however, CMR is neither feasible acutely nor widely available^1^ and is not routinely recommended in contemporary practice guidelines.^1^, ^11^

The index of microvascular resistance (IMR) is a direct invasive measure of microvascular function that can be performed routinely in the cardiac catheterization laboratory immediately after revascularization to identify patients with failed reperfusion. IMR is inversely associated with left ventricular (LV) function post‐MI^12^ and positively associated with infarct size^12^ and pathology.^2^ An IMR >40 is a multivariable associate of mortality post‐STEMI.^12^, ^13^

Because downstream microvascular resistance influences coronary blood flow, the characteristics of the thermodilution waveform in the culprit coronary artery reflect microvascular dysfunction and infarct pathology in patients with acute STEMI.^14^ In a study of 88 patients with acute STEMI, a bimodal thermodilution waveform in the culprit coronary artery was associated with microvascular dysfunction and cardiac death at 6 months after STEMI, in contrast to IMR.^14^ However, this analysis had some limitations including a modest sample size, short duration of follow‐up (6 months), and lack of follow‐up imaging. We aimed to further assess the clinical significance of the thermodilution waveform in the culprit coronary artery in a large and relatively unselected population of STEMI survivors.

## Methods

The data, analytic methods, and study materials will be made available on request to other researchers for purposes of reproducing the results or replicating the procedure. The study was approved by the West of Scotland Research Ethics Committee, reference 10‐S0703‐28, and informed consent was obtained from each patient.

### Study Population

Between July 14, 2011 and November 22, 2012, 278 STEMI patients with acute STEMI who were reperfused predominantly by emergency PCI were prospectively enrolled (British Heart Foundation MR‐MI; ClinicalTrials.gov: NCT02072850). All patients gave informed consent to undergo a diagnostic guidewire‐based assessment at the end of the PCI procedure, then CMR 2 days and 6 months later, and follow‐up for health outcomes in the longer term. Patients with a contraindication to CMR, such as a pacemaker or severe renal dysfunction, were not enrolled.

### Thermodilution in the Culprit Coronary Artery

Thermodilution curves were manually acquired after PCI of the infarct‐related artery using a dual‐sensor pressure‐ and temperature‐sensitive coronary guidewire (Abbott Vascular, Santa Clara, CA). The diagnostic wire was calibrated outside the body, equalized with aortic pressure at the ostium of the guide catheter, and then advanced to the distal third of the culprit artery. We used guide catheters without side holes to allow delivery of a saline bolus into the coronary ostium. Care was also taken to ensure that the guide catheter was properly intubated and that the catheter was flushed with saline, thereby removing contrast medium that could potentially interfere with the measurements. The injections were preceded by a 2‐mL bolus of 200 μg of nitrate.

Thermodilution curves in the culprit coronary artery were obtained by repeated manual injections of 3 mL of room‐temperature saline during maximal hyperemia induced by continuous intravenous infusion of adenosine (140 μg/[kg·min]). The average of the 3 values was taken as the mean hyperemic transit time. Following injection of the saline bolus into the coronary artery, the reduction in temperature of the coronary blood was detected by the thermistor at the distal end of the guidewire. The thermodilution curve (time [seconds] on x‐axis, temperature on y‐axis) was recorded in real time (RADIAnalyzer, Abbott Vascular, Santa Clara, CA) and available for analysis (Radiview 2.2, St Jude Medical, St. Paul, MN). IMR was calculated as the product of simultaneously measured distal coronary pressure (mm Hg) and mean hyperemic transit time (seconds), as previously described.^12^, ^13^

### Assessment of Coronary Thermodilution Waveforms

**Figure 1:**
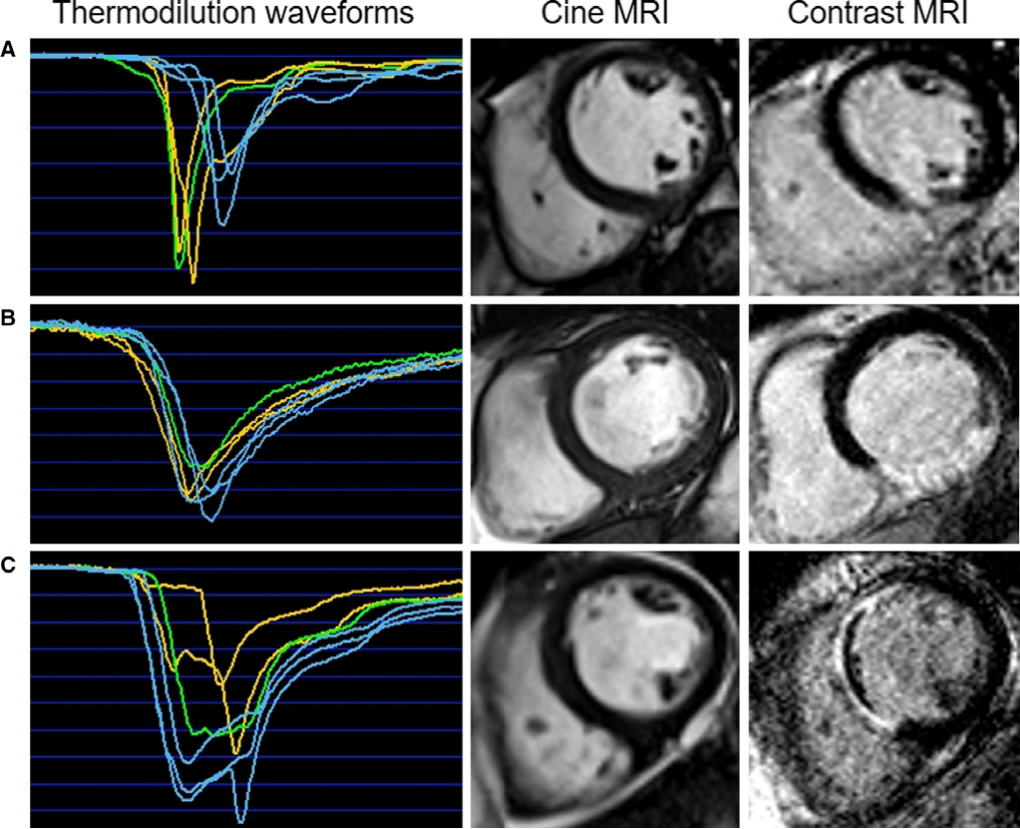
Thermodilution waveforms obtained in the culprit coronary artery at the end of PCI in 3 patients with acute STEMI. The coronary thermodilution curves are obtained in triplicate at rest (blue) and then again during hyperemia (orange) induced by intravenous infusion of adenosine (140 μg/[kg·min]). The green‐highlighted curve represents the “live” measurement of interest according to the software settings. A, A patient with a narrow unimodal waveform and the image obtained using CMR with late gadolinium enhancement (showing a subendocardial infarct with no microvascular obstruction) 2 days later; (B) a wide unimodal waveform and a larger area of transmural infarction revealed by CMR; (C) a bimodal waveform in association with an extensive area of infarction complicated by microvascular obstruction (central dark zone within the bright area of infarction). CMR indicates coronary magnetic resonance imaging; MRI, magnetic resonance imaging; PCI, percutaneous coronary intervention; STEMI, ST‐segment–elevation myocardial infarction.

Thermodilution waveforms were analyzed by a trained observer (S.N.Y.) who was blind to all of the magnetic resonance imaging (MRI) and clinical data. S.N.Y was trained and supported by D.C. and C.B. The waveforms were classified into 3 groups according to the shape of the thermodilution curve: sharp unimodal, wide unimodal, and bimodal (Figure 1). The mean transit time from the start of the thermodilution curve (reduction in temperature) to the maximum reduction in temperature of all the unimodal thermodilution curves was measured (mean [SD]=0.42 [0.15] seconds). A narrow unimodal waveform was defined as an acute temperature reduction followed by rapid return to the resting temperature, with a time from the beginning of the reduction to the minimum temperature (trough) of less than 0.42 seconds. A wide unimodal waveform was defined as a temperature decrease to a nadir followed by gradual return to the baseline temperature with a time from the inflection of the curve to the minimum temperature of more than 0.42 seconds. A bimodal waveform was defined as a waveform with 2 distinct nadirs (defined as the second nadir being lower than 20% of the peak temperature drop). Intra‐ and interobserver (S.N.Y. and D.C.) variabilities were assessed. Following completion of these analyses, the database was then closed before association with the MRI and clinical data.

### Cardiac MRI Protocol

CMR was performed on a Siemens MAGNETOM Avanto (Erlangen, Germany) 1.5‐Tesla scanner with a 12‐element phased‐array cardiac surface soil^15^ on day 2 and 6 months after reperfusion. CMR provided the reference data on LV function, pathology, and surrogate outcomes independent of the invasive tests. The images were analyzed by observers with at least 3 years of CMR experience (N.A., D.C., I.M.) and were reviewed by an experienced cardiologist (C.B.). The CMR images were assessed independently of the coronary thermodilution data.

### Infarct Size, Microvascular Obstruction, and Myocardial Hemorrhage

The presence of acute infarction was established based on abnormalities in cine wall motion, rest first‐pass myocardial perfusion, and delayed‐enhancement imaging in 2 imaging planes.^16^ Microvascular obstruction was defined as a dark zone on early gadolinium enhancement imaging 1, 3, and 5 minutes postcontrast injection that remained present within an area of late gadolinium enhancement at 15 minutes. On the T2* CMR maps, a region of reduced signal intensity within the infarcted area, with a T2* value of <20 milliseconds^17^, ^18^, ^19^, ^20^ was considered to confirm the presence of myocardial hemorrhage.

### Myocardial Edema and Salvage

The extent of myocardial edema was defined as LV myocardium with pixel values (T2) >2 standard deviations from remote myocardium.^21^, ^22^, ^23^, ^24^, ^25^, ^26^ Myocardial salvage was calculated by subtraction of percentage infarct size from percentage area at risk, as reflected by the extent of edema.^23^, ^26^, ^27^ The myocardial salvage index was calculated by dividing the myocardial salvage area by the initial area at risk.

### ECG Analysis

A 12‐lead ECG was obtained before coronary reperfusion and 60 minutes afterwards. The extent of ST‐segment resolution on the ECG assessed 60 minutes after reperfusion compared to the baseline ECG before reperfusion^28^ was expressed as complete (≥70%), incomplete (30% to <70%), or none (≤30%).

### Coronary Angiogram Acquisition and Analyses

Coronary angiograms were acquired during usual care with cardiac catheter laboratory x‐ray (Innova^®^; GE Healthcare, Chicago, IL) and information technology equipment (Centricity^®^; GE Healthcare). The angiograms were analyzed by trained observers (J.C., V.T.Y.M.) who were blinded to all other clinical and MRI data. The TIMI coronary flow grade^29^ and frame count^30^ were assessed at initial angiography and at the end of the procedure. TIMI myocardial perfusion grade^31^ was assessed at the end of the procedure (Data S1).

### Laboratory Analyses

The acquisition of blood samples for biochemical and hematologic analyses is described in Data S1.

### Predefined Health Outcomes

We predefined adverse health outcomes that are pathophysiologically linked with the natural history of myocardial infarction and LV remodeling.^31^, ^32^ The primary composite outcome was all‐cause death or a first heart failure event following the initial hospitalization (Data S1). These outcomes were independently assessed by a cardiologist who was blinded to the baseline data.

### Statistical Analyses

Continuous variables were presented as means with standard deviation if they were normally distributed. If not, they were presented as medians with interquartile range. Differences in continuous variables between groups were assessed by the Student t test or ANOVA if the data were normally distributed or by the nonparametric Mann‐Whitney test or Kruskal‐Wallis test if the data were not normally distributed. Categorical variables are expressed as the number and percentage of patients. Differences in categorical variables between groups were assessed using a Fisher test.

Twenty subjects were randomly selected from the 278 patients, and the thermodilution pattern of these 20 subjects was analyzed repeatedly. The interobserver (D.C. and S.N.Y.) and intraobserver reliability for the visual assessment of thermodilution waveforms was assessed using weighted Cohen κ. Univariable and multivariable associations were assessed using binary logistic regression or linear regression. Logistic regression was used to identify potential clinical predictors of all‐cause death or heart failure hospitalization, including patient characteristics, CMR findings, IMR, and thermodilution waveform pattern. The Akaike information criterion was used to assess the relative quality of the statistical models. All *P*‐values are 2‐sided, and a *P*‐value of more than 0.05 indicates the absence of statistically significant effect. Statistical analyses were performed using SPSS version 22 (IBM, Armonk, NY).

## Results

### Patient Characteristics

**Figure 2:**
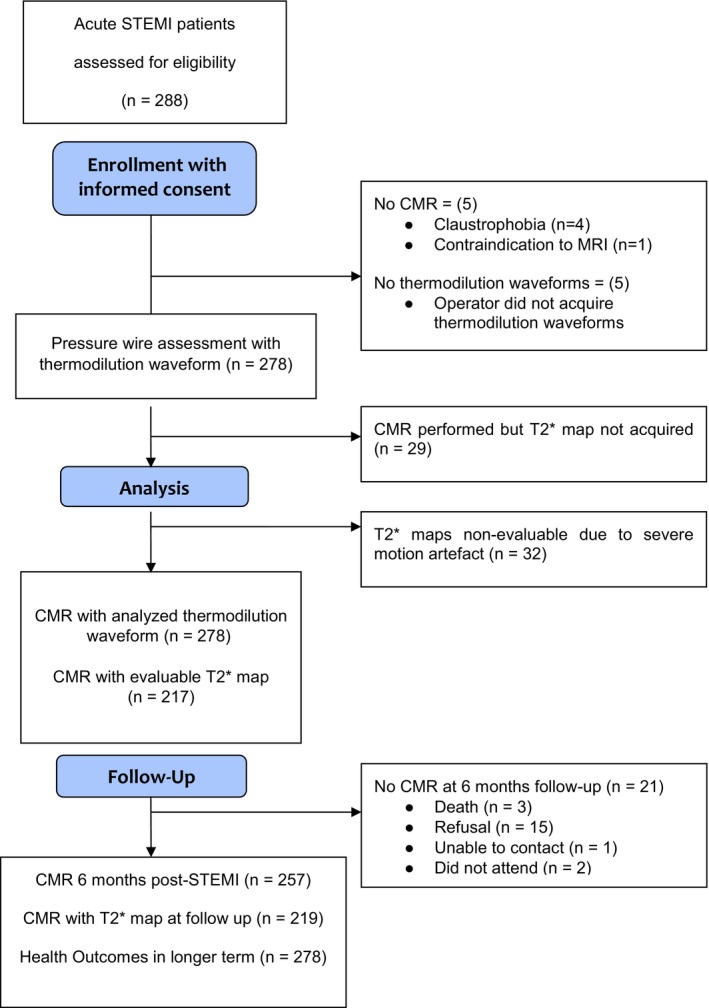
CONSORT (Consolidated Standards of Reporting Trials) flow diagram of the study. CMR indicates cardiac magnetic resonance; STEMI, ST‐segment–elevation myocardial infarction.

A total of 278 patients had thermodilution performed in the culprit coronary artery (Table 1; Figure 2). Of these, 143 (51%) had a narrow unimodal waveform, 100 (36%) patients had a wide unimodal waveform, and 35 (13%) patients had a bimodal waveform. Representative cases are illustrated in Figure 1. The intraobserver variability and interobserver variability were κ=0.740 and 0.706, respectively.

**Table 1:** Characteristics of the Patients Grouped by the Type of Thermodilution Waveform in the Culprit Coronary Artery at the End of PCI

Characteristics	All Patients n=278	Narrow Unimodal n=143 (51%)	Wide Unimodal n=100 (36%)	Bimodal n=35 (13%)	*P* Value
Age, y	59±11	58±11	61±11	62±12	0.114
Male, n (%)	199 (72)	99 (69)	76 (76)	24 (69)	0.474
BMI, kg/m^2^	29±5	29±5	29±4	28±5	0.632
Hypertension, n (%)	87 (31)	45 (32)	28 (28)	14 (40)	0.399
Current smoking, n (%)	175 (63)	95 (66)	59 (59)	21 (60)	0.451
Diabetes mellitus, n (%)^a^	32 (12)	17 (12)	12 (12)	3 (9)	0.930
Previous myocardial infarction, n (%)	18 (7)	13 (9)	3 (3)	2 (6)	0.154
Previous PCI, n (%)	14 (5)	8 (6)	4 (4)	2 (6)	0.797
Presenting characteristics					
Heart rate, bpm	78±17	78±17	77±17	80±15	0.575
Systolic blood pressure, mm Hg	135±25	135±26	136±25	132±23	0.752
Symptom onset to reperfusion, min	256±211	259±229	244±186	277±208	0.731
Killip class, n (%)					
I	198 (71)	110 (77)	72 (72)	16 (46)	0.003
II	59 (21)	26 (18)	22 (22)	11 (4)	
III/IV	21 (8)	7 (5)	6 (6)	8 (23)	
ST‐segment resolution post‐PCI, n (%)					
Complete, ≥70%	126 (46)	67 (47)	48 (48)	11 (31)	0.473
Incomplete, 30% to <70%	113 (41)	57 (40)	18 (51)	38 (38)	
None, ≤30%	38 (14)	18 (13)	14 (14)	6 (17)	
IMR>40	84 (30)	16 (11)	45 (45)	23 (66)	<0.001
Reperfusion strategy, n (%)^b^					
Primary PCI	257 (92)	135 (94)	88 (88)	34 (97)	0.369
Number of diseased arteries, n (%)					
1	153 (55)	83 (58)	50 (50)	20 (57)	0.475
2	85 (31)	45 (32)	30 (30)	10 (29)	
3	36 (13)	14 (10)	17 (17)	5 (14)	
Left main	4 (1)	1 (1)	3 (3)	0 (0)	
Culprit artery, n (%)					
Left anterior descending	101 (36)	55 (39)	31 (31)	15 (43)	0.418
Left circumflex	52 (19)	30 (21)	17 (17)	5 (14)	
Right coronary	125 (45)	58 (41)	52 (52)	15 (43)	
Culprit artery TIMI flow grade at initial angiography, n (%)					
0/1	204 (73)	93 (65)	79 (79)	32 (91)	0.007
2	48 (17)	33 (23)	12 (12)	3 (9)	
3	26 (9)	17 (12)	9 (9)	0 (0)	
Culprit artery TIMI flow grade post‐PCI, n (%)					
0/1	1 (0)	1 (1)	0 (0)	0 (0)	0.026
2	12 (4)	5 (4)	2 (2)	5 (14)	
3	265 (95)	137 (96)	98 (98)	30 (86)	
TIMI Myocardial Blush Grade					
0/1	78 (28)	38 (27)	27 (27)	13 (37)	0.436
2/3	200 (72)	105 (73)	73 (73)	22 (63)	
Treatment, n (%)					
Aspirin	277 (100)	142 (99)	100 (100)	35 (100)	1.000
Clopidogrel	275 (99)	141 (99)	99 (99)	35 (100)	1.000
β‐Blocker	266 (96)	137 (96)	95 (95)	34 (97)	0.861
ACE inhibitor or angiotensin receptor blocker	275 (99)	141 (99)	99 (99)	35 (100)	0.769
Blood results on admission					
Troponin I, ng/L, median (Q1‐Q3)	1710 (109–5104)	1628 (97–3673)	1762 (111–5245)	2932 (129–7766)	0.159
Monocytes, ×10^9^/L	1.1±0.4	1.0±0.3	1.1±0.4	1.3±0.4	0.011
NT‐proBNP, pg/mL	1299±1473	964±1218	1440±1535	2344±1848	0.006

Killip classification of heart failure after acute myocardial infarction: class I, no heart failure; class II, pulmonary rales or crepitations, a third heart sound, and elevated jugular venous pressure; class III, acute pulmonary edema; class IV, cardiogenic shock. ACE indicates angiotensin converting enzyme; BMI, body mass index; IMR, index of microvascular resistance; IQR, interquartile range; NT‐proBNP, N‐terminal pro b‐type natriuretic peptide; PCI, percutaneous coronary intervention; TIMI, Thrombolysis in Myocardial Infarction grade.

a Data are reported as mean (SD), median (IQR), or N (%) as appropriate. *P* values have been obtained from a t test (t), Mann‐Whitney test, or Fisher test.

b Reperfusion strategy includes primary PCI (n=257 [92%]), rescue PCI (failed thrombolysis; n=14 [5%]), and successful thrombolysis (n=7 [3%]). A diseased artery is defined as a stenosis >50% in a major coronary artery >2.5 mm in diameter.

The patients with a bimodal coronary thermodilution morphology had more adverse clinical characteristics, including an occluded culprit coronary artery at presentation (*P*=0.007), worse flow in the culprit artery at the end of the procedure (*P*=0.026), a higher proportion with Killip class III and IV heart failure (*P*=0.003), and a higher circulating monocyte count (*P*=0.011). A bimodal waveform was also positively associated with the circulating concentration of NT‐proBNP (N‐terminal pro b‐type natriuretic peptide) (*P*=0.006) (Table 1).

### Thermodilution Waveforms and IMR

The median (interquartile range) IMR of the bimodal morphology group was higher than those in the narrow and wide unimodal groups (narrow unimodal 18 [12‐27] units, wide unimodal 36 [21‐52] units, bimodal 54 [32‐101] units, *P*<0.001). Eighty‐four (30%) patients had an IMR >40 units. An IMR >40 occurred in 16 (11%) patients in the narrow unimodal group, 45 (45%) patients in the wide unimodal group, and 23 (66%) patients in the bimodal waveform group, respectively (*P*<0.001).

### Cardiac Magnetic Resonance Imaging

#### Thermodilution Waveforms and LV Ejection Fraction

The CMR findings at baseline and 6 months were grouped by the type of culprit artery thermodilution waveform (Table 2). The LV ejection fractions at baseline (*P*<0.001) and at follow‐up (*P*=0.001) were lower in the bimodal group.

**Table 2:** CMR Findings at Baseline and at 6 Months in STEMI Patients Grouped by Thermodilution Waveforms Post‐PCI

Characteristics^a^	All Patients (n=278)	Thermodilution Waveform			*P* Value
		Narrow Unimodal (n=143)	Wide Unimodal (n=100)	Bimodal (n=35)	
CMR findings 2 d post‐MI					
LV ejection fraction, %	55±10	57±8	55±10	49±12	<0.001
LV end‐diastolic volume, mL					
Men	160±32	156±32	164±34	166±25	0.204
Women	125±24	128±25	123±28	121±27	0.902
LV end‐systolic volume, mL					
Men	74±26	69±22	77±29	88±25	0.003
Women	55±17	54±17	53±18	61±19	0.437
LV mass, g					
Men	144±33	144±33	144±36	142±19	0.965
Women	99±22	97±14	102±30	101±27	0.682
Edema and infarct characteristics					
Myocardial edema, % LV mass	32±12	30±11	33±11	36±13	0.009
Infarct size, % LV mass	18±13	16±13	18±14	25±15	0.003
Myocardial salvage, % LV mass	19±9	19±9	19±8.3	19±9	0.881
Myocardial salvage index, % LV mass	63±24	65±24	61±23	56±24	0.115
Microvascular obstruction present, n (%)	162 (58)	75 (52)	58 (58)	29 (83)	0.005
Microvascular obstruction, % LV mass	3±5	2±4	3±5	6±8	<0.001
Presence of myocardial hemorrhage	90 (42)	40 (35)	32 (42)	18 (67)	0.011
Myocardial hemorrhage, % LV mass	8.3±6.2	8.1±6.7	7.0±5.8	11.2±5.1	0.070
CMR findings 6 mo post‐MI (n=257)					
LV ejection fraction at 6 mo, %	62±10	64±9	62±9	57±12	0.001
LV end‐diastolic volume at 6 mo, mL					
Men	167±42	161±36	171±47	186±47	0.035
Women	127±29	128±25	128±37	121±27	0.799
LV end‐systolic volume at 6 mo, mL					
Men	67±35	61±24	69±40	88±47	0.004
Women	46±17	46±19	44±13	49±20	0.721
Infarct size at 6 mo					
Infarct size, % LV mass	13±10	11±9	13±10	17±12	0.004

Data are given as n (%) or mean (SD). *P*‐values were obtained from a t test (t), a Mann‐Whitney test, or a Fisher test. CMR indicates cardiac magnetic resonance imaging; IQR, interquartile range; LV, left ventricle; MI, myocardial infarction; PCI, percutaneous coronary intervention; STEMI, ST‐segment–elevation myocardial infarction.

a Data are reported as mean (SD), median (IQR), or n (%) as appropriate.

#### Thermodilution Waveforms and Infarct Characteristics

Acute and final infarct sizes at 6 months were greatest in the bimodal waveform group (Table 2). Microvascular obstruction and myocardial hemorrhage were associated with thermodilution waveform pattern (*P*=0.002 and 0.004, respectively) (Table 3), and proportionately more patients in the bimodal waveform group were affected (Table 2).

**Table 3:** Univariable Association Among Thermodilution Waveform, IMR (for a 5‐Unit Change), and LV Function and Pathology Revealed on CMR

Left Ventricular Function and Pathology Revealed on CMR	Microvascular Obstruction	Intramyocardial Hemorrhage	Infarct Size During Follow‐Up at 6 Months	Change in LV Ejection Fraction	Change in LV End‐Diastolic Volume
	OR (95% CI) *P* Value	OR (95% CI) *P* Value	Coefficient (95% CI) *P* Value	Coefficient (95% CI) *P* Value	Coefficient (95% CI) *P* Value
IMR (for a 5‐unit change)	1.12 (1.06, 1.18) <0.001	1.11 (1.05, 1.17) <0.001	0.63 (0.44, 0.82) <0.001	−6.78 (−5.33, −8.24) <0.001	0.85 (0.31, 1.40) 0.002
IMR>40 (yes vs no)	2.28 (1.34, 3.89) 0.002	2.26 (1.24, 4.05) 0.006	1.56 (0.24, 2.88) 0.020	−1.83 (−3.80, 0.14) 0.068	9.97 (2.88, 17.07) 0.006
Thermodilution waveform (reference=narrow unimodal)					
Wide unimodal	1.25 (0.75, 2.10) 0.392	1.35 (0.74, 2.44) 0.392	2.24 (−0.37, 4.86) 0.092	−0.18 (−2.26, 1.91) 0.869	4.64 (−2.44, 11.72) 0.198
Bimodal	4.38 (1.72, 11.20) 0.002	3.70 (1.52, 8.99) 0.004	6.38 (2.54, 10.22) 0.001	−0.50 (−3.60, 2.59) 0.749	7.69 (−2.81, 18.18) 0.769
IMR >40 and bimodal	2.44 (2.63, 49.84) 0.001	1.50 (1.56, 12.98) 0.005	1.15 (−1.07, 3.38) 0.309	−4.97 (−8.31, −1.64) 0.004	7.37 (−5.07, 19.81) 0.244

CI indicates confidence interval; CMR, cardiac magnetic resonance imaging; IMR, index of microvascular resistance; LV, left ventricle; OR, odds ratio.

### Multivariable Associations of Thermodilution Waveform Type in the Culprit Coronary Artery and Microvascular Infarct Pathology

#### Microvascular Obstruction

In a binary logistic regression model with baseline characteristics, thermodilution waveform status was a multivariable associate of microvascular obstruction revealed by CMR 2 days post–myocardial infarction (Table 4). This relationship was no longer significant when IMR>40 or an IMR (5 per unit) were included.

**Table 4:** Multivariable Associations Between Clinical Characteristics at Presentation, Including Thermodilution Waveforms, and the Occurrence of Microvascular Obstruction 2 Days Later in Patients With Acute STEMI

Binary Logistic Regression	Odds Ratio (95% CI)	*P* Value
Bimodal waveform	5.29 (1.73, 16.22)	0.004
ST‐segment resolution		
None	2.44 (1.30, 4.57)	0.006
Partial	3.54 (1.34, 9.33)	0.011
Sex	2.17 (1.13, 4.16)	0.020
Previous PCI	10.97 (1.03, 116.83)	0.047

Other baseline characteristics included are smokers (*P*=0.114), hypertension (*P*=0.155), wide unimodal waveform (*P*=0.388), hypercholesterolemia (*P*=0.390), previous angina (*P*=0.405), age, y (*P*=0.599), TIMI Myocardial Perfusion Grade post‐PCI 2/3 (*P*=0.60), BMI, kg/m^2^ (*P*=0.615), SBP per 10 mm Hg (*P*=0.685), previous MI (*P*=0.776), heart rate, bpm (*P*=0.819), diabetes mellitus (*P*=0.980), symptoms to reperfusion time per 10 minutes (*P*=0.084). BMI indicates body mass index; CI, confidence interval; MI, myocardial infarction; PCI, percutaneous coronary intervention; SBP, systolic blood pressure; STEMI, ST‐segment–elevation myocardial infarction; TIMI, Thrombolysis in Myocardial Infarction.

#### Myocardial Hemorrhage

Thermodilution waveform status was a multivariable associate of myocardial hemorrhage (Table 5), but this relationship was no longer significant when an IMR >40 (*P*>0.05) or an IMR (per 5 unit) (*P*>0.05) was included.

**Table 5:** Multivariable Associations Between Clinical Characteristics at Presentation, Including Thermodilution Waveform, and the Occurrence of Myocardial Hemorrhage 2 Days Later in Patients With Acute STEMI

Binary Logistic Regression	Odds Ratio (95% CI)	*P* Value
Smoker	3.93 (1.70, 8.65)	0.001
ST‐segment resolution—partial	3.88 (1.36, 11.09)	0.012
Sex	2.65 (1.20, 5.85)	0.016
Bimodal waveform	3.45 (1.16, 10.26)	0.026

Other baseline characteristics included are ST‐segment resolution—none (*P*=0.066), previous PCI (*P*=0.086), symptoms to reperfusion time per 10 minutes (*P*=0.171), wide unimodal waveform (*P*=0.171), age, y (*P*=0.218), hypercholesterolemia (*P*=0.254), heart rate, bpm (*P*=0.545), previous MI (*P*=0.556), hypertension (*P*=0.573), systolic blood pressure per 10 mm Hg (*P*=0.582), percentage residual stenosis (*P*=0.773), BMI (*P*=0.860), diabetes mellitus (*P*=0.861), previous angina (*P*=0.870), TIMI Myocardial Perfusion Grade post‐PCI 2/3 (*P*=0.976). BMI indicates body mass index; CI, confidence interval; MI, myocardial infarction; PCI, percutaneous coronary intervention; STEMI, ST‐segment–elevation myocardial infarction; TIMI, Thrombolysis in Myocardial Infarction.

### Thermodilution Waveforms and LV Outcomes During Follow‐Up

#### Changes in LV End‐Diastolic Volume

IMR (5 unit difference) was a multivariable associate of change in LV end‐diastolic volume, independent of thermodilution waveform status (regression coefficient [95% confidence interval]=0.87 [0.11, 1.62], *P*=0.024) (Table S1).

#### Changes in LV Ejection Fraction

Thermodilution waveform status, IMR>40, and IMR (for a 5‐unit change) were not associates of changes in LV ejection fraction (Table S2).

### Thermodilution Waveforms and Longer‐Term Health Outcomes

#### All‐Cause Death or Heart Failure Hospitalization

The median duration of follow‐up was 1469 days in 278 STEMI patients. During this time, 40 (14.4%) participants died or experienced a heart failure episode. The occurrence of a bimodal thermodilution waveform was independently associated with all‐cause death and heart failure hospitalization (odds ratio [95% confidence interval]=2.70 [1.10, 6.63]; *P*=0.031), independent of IMR>40, ST‐segment resolution, and TIMI Myocardial Perfusion grade (Table 6). However, the relationship was not statistically significant (odds ratio [95% confidence interval]=2.35 [0.90, 6.14]; *P*=0.080) when infarct size was included in the model (Table 6).

**Table 6:** Association Between Clinical Factors at the Time of Invasive Management, Including the Type of Coronary Thermodilution Waveform, and All‐Cause Death or Heart Failure Hospitalization and Death Only After the Index Admission During Longer‐Term Follow‐Up in 278 STEMI Patients

Associations	Odd Ratio (95% CI)	*P* Value
Univariable associations with all‐cause death or heart failure		
Bimodal waveform	4.01 (1.80, 8.93)	0.001
IMR>40 and bimodal waveform	5.77 (2.33, 14.31)	<0.001
IMR>40	2.41 (1.22, 4.77)	0.012
IMR (for a 5‐unit change)	1.07 (1.02, 1.12)	0.003
TIMI Myocardial PerfusionGrade 2/3	1.67 (0.83, 3.36)	0.154
ST‐segment resolution<50%	2.52 (1.23, 5.17)	0.011
Acute infarct size	1.09 (1.06, 1.12)	<0.001
Multivariable associations with all‐cause death or heart failure		
Model A—bimodal waveform and IMR		
Bimodal waveform	2.46 (0.95, 6.40)	0.065
IMR (for a 5‐unit change)	1.04 (0.99, 1.10)	0.144
ST‐segment resolution<50%	1.92 (0.90, 4.10)	0.093
TIMI Myocardial Perfusion Grade 2/3	1.28 (0.58, 2.85)	0.545
Akaike Information Criterion		131.84
Model B—bimodal waveform and IMR>40		
Bimodal waveform	2.70 (1.10, 6.63)	0.031
IMR>40	1.84 (0.84, 4.03)	0.128
ST‐segment resolution<50%	1.92 (0.90, 4.10)	0.092
TIMI Myocardial Perfusion Grade 2/3	1.32 (0.60, 2.90)	0.487
Akaike Information Criterion		51.60
Model C—bimodal waveform only		
Bimodal waveform	3.32 (1.41, 7.82)	0.006
ST‐segment resolution<50%	2.01 (0.94, 4.26)	0.329
TIMI Myocardial Perfusion grade 2/3	1.47 (0.68, 3.16)	0.070
Akaike Information Criterion		33.28
Model D—IMR (for a 5‐unit change) only		
IMR (for a 5‐unit change)	1.07 (1.01, 1.12)	0.014
ST‐segment resolution <50%	2.08 (0.99, 4.38)	0.054
TIMI Myocardial Perfusion Grade 2/3	1.27 (0.58, 2.78)	0.558
Akaike Information Criterion		199.91
Model E—IMR>40 only		
IMR>40	2.29 (1.09, 4.81)	0.028
ST‐segment resolution <50%	2.14 (1.02, 4.49)	0.043
TIMI Myocardial Perfusion Grade 2/3	1.36 (0.63, 2.93)	0.435
Akaike Information Criterion		34.92
Model F—IMR>40 with bimodal waveform only		
IMR>40 and bimodal waveform	4.47 (1.68, 11.89)	0.003
ST‐segment resolution<50%	2.02 (0.95, 4.31)	0.068
TIMI Myocardial Perfusion grade 2/3	1.34 (0.61, 2.93)	0.468
Akaike Information Criterion		32.16
Model G—Bimodal waveform and acute infarct size		
Bimodal waveform	2.35 (0.90, 6.14)	0.080
Acute infarct size	1.08 (1.04, 1.11)	<0.001
ST‐segment resolution<50%	1.24 (0.54, 2.89)	0.611
TIMI Myocardial Perfusion Grade 2/3	1.27 (0.55, 2.92)	0.577
Akaike Information Criterion		181.66
Model H—IMR (for a 5‐unit change) and infarct size		
IMR (for a 5‐unit change)	1.02 (0.96, 1.08)	0.563
Acute infarct size	1.08 (1.04, 1.11)	<0.001
ST‐segment resolution<50%	1.41 (0.63, 3.18)	0.405
TIMI Myocardial Perfusion Grade 2/3	1.26 (0.53, 2.95)	0.602
Akaike Information Criterion		182.85
Model I—IMR>40 with bimodal waveform and infarct size		
IMR>40 and bimodal waveform	2.65 (0.91, 7.74)	0.074
Acute infarct size	1.08 (1.04, 1.11)	<0.001
ST‐segment resolution<50%	1.31 (0.57, 3.00)	0.525
TIMI Myocardial Perfusion Grade 2/3	1.21 (0.52, 2.82)	0.655
Akaike Information Criterion		177.15
Univariable associations with all‐cause death only		
Acute infarct size	1.04 (1.01, 1.08)	0.009
Bimodal waveform	1.33 (0.37, 4.82)	0.664
IMR >40	1.07 (0.39, 2.92)	0.893
IMR (for a 5‐unit change)	0.99 (0.92, 1.08)	0.890
IMR>40 and bimodal waveform	1.33 (0.29, 6.17)	0.713

Median duration of follow‐up was 4 years. Minimum to maximum postdischarge censor duration was 1 to 1800 days.

All‐cause death or heart failure occurred in 40 (14.4%) patients. Death occurred in 19 (6.8%) patients. Logistic regression was used to explore the associations. CI indicates confidence interval; IMR, index of microvascular resistance; STEMI, ST‐segment–elevation myocardial infarction; TIMI, Thrombolysis in Myocardial Infarction.

Nineteen (6.8%) patients died during follow‐up. IMR alone was not associated with death (Table 6).

## Discussion

The main findings of our study are these: (1) thermodilution in the culprit coronary artery was straightforward and feasible to perform in a comparatively large number of patients with acute STEMI, and intraobserver and interobserver variabilities for classification of the waveform morphologies were reasonably high; (2) thermodilution waveform type was associated with IMR. A bimodal waveform was a multivariable associate of microvascular obstruction; however, this relationship was no longer significant when IMR was included (either IMR [for a 5‐unit change] or IMR>40); (3) IMR (for a 5‐unit change) was an independent predictor of myocardial hemorrhage; (4) a bimodal thermodilution waveform was a multivariable associate of adverse clinical outcomes during follow‐up, including after adjustment for IMR>40.

Taken together, these results indicate that a bimodal waveform is an associate of adverse clinical outcomes post–myocardial,infarction and may be useful for risk stratification of STEMI patients immediately after reperfusion therapy. Waveform classification and IMR have relative merits. The waveform classification represents a binary approach to risk stratification, whereas IMR and other indices (such as hyperemic microvascular resistance derived using Doppler) provide a continuous measure of microvascular dysfunction and in this sense may be more informative.

Fearon et al first reported that an IMR>40 measured after angiographically successful primary PCI was an independent predictor of adverse clinical outcome.^12^ We previously found that an IMR value of >27 was most closely associated with microvascular obstruction and myocardial hemorrhage, whereas an IMR >40 was most closely associated with all‐cause death or heart failure.^13^ We also observed that IMR was associated with the systemic concentration of IL‐6 on the first day post‐STEMI, reflecting systemic inflammation and vascular injury.^13^ The current study extends these findings because a bimodal thermodilution waveform was associated with heart failure post–myocardial infarction, systemic inflammation (monocyte count), and circulating concentrations of NT‐proBNP early post–myocardial infarction.

Fukunaga et al^14^ reported that a bimodal thermodilution waveform was independently associated with the presence of microvascular obstruction on CMR, and this was associated with worse midterm clinical outcomes. They hypothesized that the bimodal thermodilution waveform may be explained by resistance to antegrade flow within the culprit coronary artery due to microvascular destruction.^14^ IMR derived from a bimodal curve incorporates a mean transit time derived from disordered antegrade coronary flow, potentially even transient retrograde flow, secondary to microvascular dysfunction.^33^ This scenario calls into question the validity of transit time as a proxy for flow when the thermodilution curve is bimodal. On the other hand, the prognostic associations for IMR in continuous and binary forms are well established. Our observations are in keeping with those of Fukunaga et al^14^ and suggest that the bimodal thermodilution waveform reflects more severe, persistent microvascular injury and has the potential for immediate risk stratification in the catheterization laboratory. Patients with a bimodal waveform are at risk of adverse outcomes, indicating the need for more intensive therapy and follow‐up.

In our study, microvascular obstruction and myocardial hemorrhage were associated with thermodilution waveform pattern, and each pathology was more prevalent in patients with a bimodal morphology; however, this relationship was not independent of other characteristics when assessed in a multivariable regression model. Instead, our results showed that IMR (for a 5‐unit change) was a stronger associate of microvascular obstruction and myocardial hemorrhage. Logistic regression analysis showed that bimodal thermodilution waveform status was a stronger predictor of all‐cause death and heart failure hospitalization than IMR. When infarct size measured on CMR 2 days later was included, the prognostic significance of IMR was lost. IMR (for a 5‐unit change) was the only independent predictor of infarct size during follow‐up on multivariate analyses (Table S3). The associations among IMR (ordinal value), thermodilution waveform status (bimodal), and myocardial hemorrhage, reflecting severe irreversible vascular damage within the infarct zone, provide a pathophysiological basis for adverse health outcomes in the longer term.

Our study extends that of Fukunaga et al.^14^ Some of the differences in the results may relate to differences in the patient populations, sample size, CMR methods used, and duration of follow‐up. For example, Fukunaga et al^14^ excluded STEMI patients presenting with Killip class III/IV acute heart failure and left mainstem culprit lesions.^14^ The only exclusion criterion in our study was a contraindication to contrast MRI. In addition, our study was 3 times larger and had substantially longer follow‐up (median of 1469 days versus 6 months). The intra‐ and interobserver coefficients reported in our study are lower than those reported by Fukunaga et al,^14^ implying that development of an automated algorithm may enhance precision and accuracy in the clinic.

Similar to IMR (for a 5‐unit change), a bimodal thermodilution waveform is a stronger predictor of adverse clinical outcome than ST‐segment resolution on ECG, or angiographic flow grades and may be useful for risk stratification of STEMI patients immediately after reperfusion. IMR is independently associated with microvascular obstruction and myocardial hemorrhage, and because it is a continuous value, it holds the potential to quantify the efficacy of novel reperfusion therapies designed to restore microvascular perfusion and limit infarct size. This possibility is currently being assessed in a randomized, controlled phase 2 clinical trial of low‐dose alteplase (10 and 20 mg) or placebo directly administered into the culprit coronary artery after reperfusion but before stenting (T‐TIME ClinicalTrials.gov, Identifier: NCT02257294).

## Conclusion

Our study adds to previous investigations using coronary thermodilution for risk assessment in patients with acute STEMI. We conclude that a bimodal thermodilution waveform identifies high‐risk patients who may benefit from more intensive follow‐up and medical therapy.

### Limitations

Our study took place in a single center, and further research in other hospitals is warranted. Only 13% of patients had a bimodal waveform, limiting to some extent its clinical impact. The waveform classification was undertaken post hoc using a core laboratory approach. The diagnostic accuracy of clinician‐reported waveform classification during real‐world practice merits further assessment.

## Sources of Funding

This work was supported by the British Heart Foundation Centre of Research Excellence Award (RE/13/5/30177), the British Heart Foundation Project Grant PG/11/2/28474, the National Health Service, and the Chief Scientist Office. Professor Berry was supported by a Senior Fellowship from the Scottish Funding Council.

## Disclosures

Siemens Healthcare provided work‐in‐progress imaging methods. Based on an institutional agreement with the University of Glasgow, Professor Berry has acted as a consultant to Abbott Vascular. Professor Oldroyd has acted as consultant to Abbott Vascular. These companies had no involvement in the current research or the article. The remaining authors have no disclosures to report.

## Supporting information


**Data S1.** Supplemental Methods
**Table S1.** Multivariable Associations Between Clinical Characteristics at Presentation, Including Thermodilution Waveforms, IMR (for a 5‐Unit Change), and the Change in LV End‐Diastolic Volume at 6 Months in Patients With Acute STEMI
**Table S2.** Multivariable Associations Between Clinical Characteristics at Presentation, Including Thermodilution Waveform, IMR (for a 5‐Unit Change), and the Change in LV Ejection Fraction at 6 Months in Patients With Acute STEMI
**Table S3.** Multivariable Associations Between Clinical Characteristics at Presentation, Including Thermodilution Waveforms, IMR (for a 5‐Unit Change), and Infarct Size 6 Months Later in Patients With Acute STEMI
